# Impact of the Type of First Medical Contact within a Guideline-Conform ST-Elevation Myocardial Infarction Network: A Prospective Observational Registry Study

**DOI:** 10.1371/journal.pone.0156769

**Published:** 2016-06-03

**Authors:** Roman Pfister, Samuel Lee, Kathrin Kuhr, Frank Baer, Wolfgang Fehske, Hans-Wilhelm Hoepp, Stephan Baldus, Guido Michels

**Affiliations:** 1 Department III of Internal Medicine, University of Cologne, Cologne, Germany; 2 Institute of Medical Statistics, Informatics and Epidemiology, University of Cologne, Cologne, Germany; 3 St. Antonius Hospital, Cologne, Germany; 4 St. Vinzenz Hospital, Cologne, Germany; KRH Robert Koch Klinikum Gehrden, GERMANY

## Abstract

**Aims:**

The impact of type of first medical contact (FMC) in the setting of a guideline conform metropolitan ST-elevation myocardial infarction (STEMI) network providing obligatory primary percutaneous coronary intervention (PCI) is unclear.

**Methods and Results:**

3,312 patients were prospectively included between 2006 and 2012 into a registry accompanying the “Cologne Infarction Model” STEMI network, with 68.4% primarily presenting to emergency medical service (EMS), 17.6% to non-PCI-capable hospitals, and 14.0% to PCI-capable hospitals. Median contact-to-balloon time differed significantly by FMC with 89 minutes (IQR 72–115) for EMS, 107 minutes (IQR 85–148) for non-PCI- and 65 minutes (IQR 48–91) for PCI-capable hospitals (p < 0.001). TIMI-flow grade III and in-hospital mortality were 75.7% and 10.4% in EMS, 70.3% and 8.6% in non-PCI capable hospital and 84.4% and 5.6% in PCI-capable hospital presenters, respectively (p both < 0.01). The association of FMC with in-hospital mortality was not significant after adjustment for baseline characteristics, but risk of TIMI-flow grade < III remained significantly increased in patients presenting to non-PCI capable hospitals.

**Conclusion:**

Despite differences in treatment delay by type of FMC in-hospital mortality did not differ significantly. The increased risk of TIMI-flow grade < III in patients presenting to non PCI-capable hospitals needs further study.

## Introduction

For patients with acute ST-elevation myocardial infarction (STEMI) rapid reperfusion of the infarct related artery is the main therapeutic goal. Delays in time to reperfusion lead to higher mortality rates regardless of way of reperfusion [1, 2]. Primary percutaneous coronary intervention (PCI) represents the preferred treatment of reperfusion over fibrinolysis [3]. However, it is a logistic challenge for regional health care systems to timely transfer STEMI patients to primary PCI since PCI is only beneficial compared with fibrinolysis when delay to reperfusion can be held below 120 minutes [4]. Accordingly, current guidelines recommend keeping the time between first medical contact (FMC) to inflation of balloon to less than 90 minutes [5, 6]. To achieve these treatment goals both metropolitan and rural communities are encouraged to establish regional systems of STEMI care that involve emergency medical services (EMS) and local hospitals with the aim of optimizing timely diagnosis of STEMI patients and direct transfer to PCI capable hospitals [7].

One of the main determinants of patient pathways in a STEMI network is the site of FMC. Patients presenting first to EMS in the field, to a hospital without a cardiac catheterization laboratory and to a hospital with PCI capability, respectively, require different logistics to ensure rapid coronary reperfusion. Although the prognostic relevance of time delay to reperfusion in STEMI patients not primarily presenting to PCI-capable hospitals in general is well established, so far the differences in system related delay to reperfusion by site of FMC and the impact on short-term outcome within an existing STEMI network implemented according to current guidelines is unclear. Identification of potential weaknesses within logistic systems is crucial to further improve STEMI care. Here, we examined the association between the site of FMC and delay to reperfusion, success of reperfusion by primary PCI and in-hospital mortality in patients treated within the Cologne Infarction Model (‘Kölner Infarkt Modell’, KIM) from 2006 to 2012, a metropolitan STEMI network implemented to provide primary PCI to all patients presenting with STEMI within recommended treatment goals.

## Material and Methods

### Cologne myocardial infarction network (‘Kölner Infarkt Modell’, KIM)

We have previously reported the setup of KIM and quality measures of STEMI care achieved in the first year of implementation [8]. Briefly, the city of Cologne has a population of about 1 million citizens and covers an area of 400 km^2^ with a maximum diameter of 28 km. The constituents of KIM are comprised of the city of Cologne’s EMS, 11 hospitals without and 5 hospitals with a catheterization laboratory available 24 hours a day and 7 days a week. KIM was developed in accordance to ESC guidelines implementing recommended processes such as training and equipment of ambulance teams, design of a regionally adapted network joining all involved health care stakeholders, sufficient and experienced centers delivering 24/7 service for primary PCI, continuous documentation of parameters of quality control and a common STEMI management protocol for all involved affiliations including pathways ensuring bypass of non-PCI capable hospitals and emergency departments with direct transfer to the catheterization laboratory [5, 8]. Notably, strategies shown to reduce system related treatment delay like activation phone call of the catheterization team by EMS was also implemented in KIM [8, 9]. The diagnosis of STEMI was made by an EMS or emergency department physician based on a 12-lead ECG in all patients according to recent definitions and this diagnosis qualified for treatment within KIM and inclusion into the KIM registry (S1 Fig) [10]. Status of cardiogenic shock was defined by the physician at first contact based on clinical assessment, heart rate and blood pressure. Initial treatment of the patient either by the EMS or the first admitting hospital included 5,000 IE of intravenous heparin, 500 mg of aspirin, and 600 mg of clopidogrel,

### Data collection

Patients who were triaged and treated according to the KIM protocol received a file consisting of documentation of FMC, treatment by EMS, treatment by hospital without PCI capability, protocol of PCI, and post-PCI treatment by discharging hospital. A secondary survey of patient hospital documentation was done to ensure completion of KIM protocols. Anonymized data was then entered into an electronic database and statistically analyzed. The study complies with the Declaration of Helsinki; the research protocol was approved by the locally appointed ethics committee of the University Hospital of Cologne and written informed consent was obtained from all patients.

### Statistical analysis

Patients registered between January 1, 2006, and December 31, 2012 were included in the analysis set if FMC was documented and the patient either died before arriving at PCI-capable hospital or treatment by PCI-capable hospital was documented. Data were described using mean values ± standard deviation (sd), median [interquartile range (IQR)], or frequencies and percentages. Differences between the three groups defined by FMC were investigated using one-way analysis of variance (ANOVA), Kruskal-Wallis test and Fisher’s exact test. The impact of FMC on postprocedural TIMI flow grade < 3 and in-hospital mortality was analyzed using univariate and multivariable logistic regression models. Variables considered in the adjusted models were baseline characteristics (age, gender, history of stroke, heart rate > 100/min, systolic blood pressure < 100 mmHg, cardiogenic shock) as well as relevant time intervals [symptom-to-contact time (log scale), contact-to-balloon time (log scale)]. As results odds ratios (OR), corresponding 95% confidence intervals (CI) and p-values (Wald test) were given. We also report results for type of FMC and contact to balloon time for subgroups of gender, age (< 75 years versus ≥ 75 years), symptom to contact time (≤ 2 hours versus > 2 hours), presence of cardiogenic shock and years of inclusion (2006 to 2009 versus 2010 to 2012) and calculated tests of interaction for these subgroups on the effect of FMC on in-hospital mortality and post-procedural TIMI flow grade < III. All reported p-values are two-sided and p-values < 0.05 were considered statistically significant. Due to the exploratory character of this study we did not adjust for multiple testing. Statistical analyses were performed using IBM SPSS Statistics for Windows, Version 22.0. Armonk, NY: IBM Corp.

## Results

### Characteristics of patients

**Fig 1:**
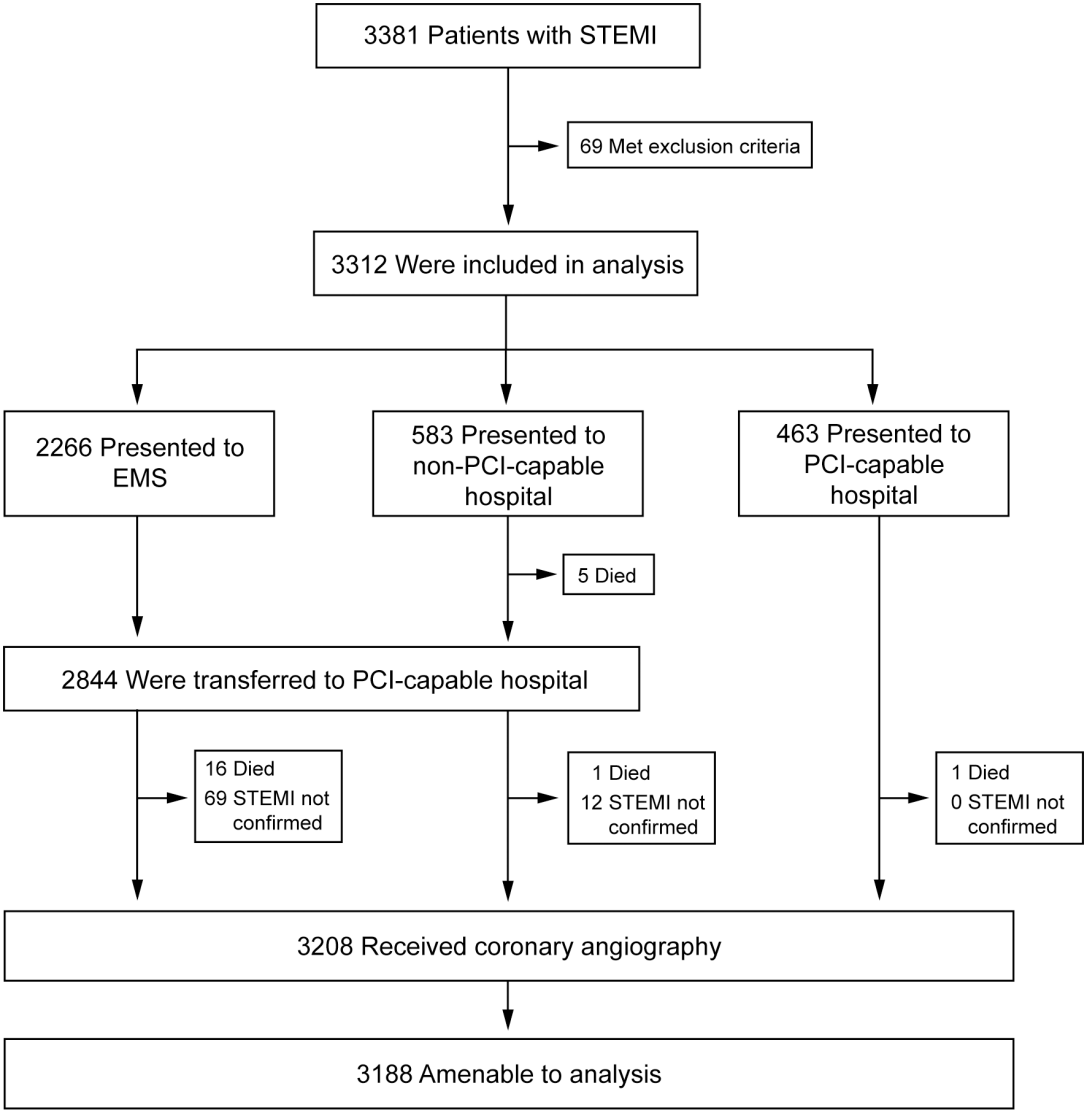
Enrollment and patient pathways. All patients with ST-segment elevation myocardial infarction (STEMI) recorded in the KIM registry were considered for inclusion in the analysis. Exclusion criteria included missing documentation of first medical contact. Analysis was done after stratification of patients according to FMC; patients presented to emergency medical services (EMS), hospitals with and without percutaneous coronary intervention (PCI) capability.

Between January 1, 2006, and December 31, 2012, 3,381 patients with STEMI were recorded in the KIM registry (Fig 1) of whom 69 patients were excluded from the analysis due to missing or implausible record of FMC. Of 3,312 included patients, 2,266 (68.4%) first presented to EMS, 583 (17.6%) to non-PCI-capable hospitals, and 463 (14.0%) directly to PCI-capable hospitals. Of the 23 (0.7%) patients who died before coronary angiography could be performed 16 (0.7%) presented to EMS, 6 (1.0%) to non-PCI-capable hospitals, and 1 (0.2%, p = 0.30) to a PCI-capable hospital. For a total of 81 patients ST-segment elevation or left bundle branch block diagnosed by the physician during first contact were not verified at the coronary intervention center; 3,208 (96.9%) patients received coronary angiography with 20 patients not amenable for analysis due to missing or incomplete record on coronary angiography.

Baseline characteristics of patients are shown in Table 1. There were significant differences across groups regarding age and parameters of hemodynamic instability as well as rate of incorrect ECG diagnosis and coronary angiography.

**Table 1:** Baseline characteristics of patients.

	Total (n = 3312)	EMS (n = 2266)	Non-PCI hospital (n = 583)	PCI hospital (n = 463)	P Value
**Age—yr**	63.2 ± 13.9	63.8 ± 13.6	63.1 ± 15.0	60.9 ± 14.0	<0.001^a^
**≥ 75 yr—no. (%)**	745 (22.5)	523 (23.1)	140 (24.0)	82 (17.7)	0.03^b^
**Male sex—no. (%)**	2443 (73.8)	1680 (74.1)	412 (70.7)	351 (75.8)	0.12^b^
**Previous stroke—no. (%)**	140 (4.2)	90 (4.0)	33 (5.7)	17 (3.7)	0.17^b^
**Heart rate > 100 bpm—no. (%)**	666 (20.1)	494 (21.8)	108 (18.5)	64 (13.8)	<0.001^b^
**Systolic blood pressure < 100 mmHg—no. (%)**	661 (20.0)	525 (23.2)	77 (13.2)	59 (12.7)	<0.001^b^
**Cardiogenic shock—no. (%)**	473 (14.3)	394 (17.4)	44 (7.5)	35 (7.6)	<0.001^b^
**Resuscitation—no. (%)**	394 (11.9)	343 (15.1)	34 (5.8)	17 (3.7)	<0.001^b^
**ASS/Clopidogrel—no. (%)**	3199 (96.6)	2181 (96.2)	564 (96.7)	454 (98.1)	0.141^b^
**Heparin—no. (%)**	3199 (96.6)	2181 (96.2)	563 (96.6)	455 (98.3)	0.077 ^b^
**Death before angiography—no (%)**	23 (0.7)	16 (0.7)	6 (1.0)	1 (0.2)	0.30^b^
**Incorrect initial ECG diagnosis of LBBB/STE—no. (%)**	81 (2.5)	69 (3.1)	12 (2.1)	0 (0)	<0.001^b^
**Angiography performed—no. (%)**	3188 (96.3)	2168 (95.7)	559 (95.9)	461 (99.6)	<0.001^b^
**Echocardiographic ejection fraction >55%**	835 (53.7)	573 (52.8)	94 (53.4)	168 (57.3)	0.44 ^b^
**41–55%**	469 (30.2)	326 (30.0)	56 (31.8)	87 (29.7)	
**< = 40%**	250 (16.1)	186 (17.1)	26 (14.8)	38 (13.0)	

LBBBB: left-bundle branch block, STE: ST-elevation. Plus-minus values are means ± SD;

^a^ One-way analysis of variance;

^b^ Fisher’s exact test

### Time intervals

**Fig 2:**
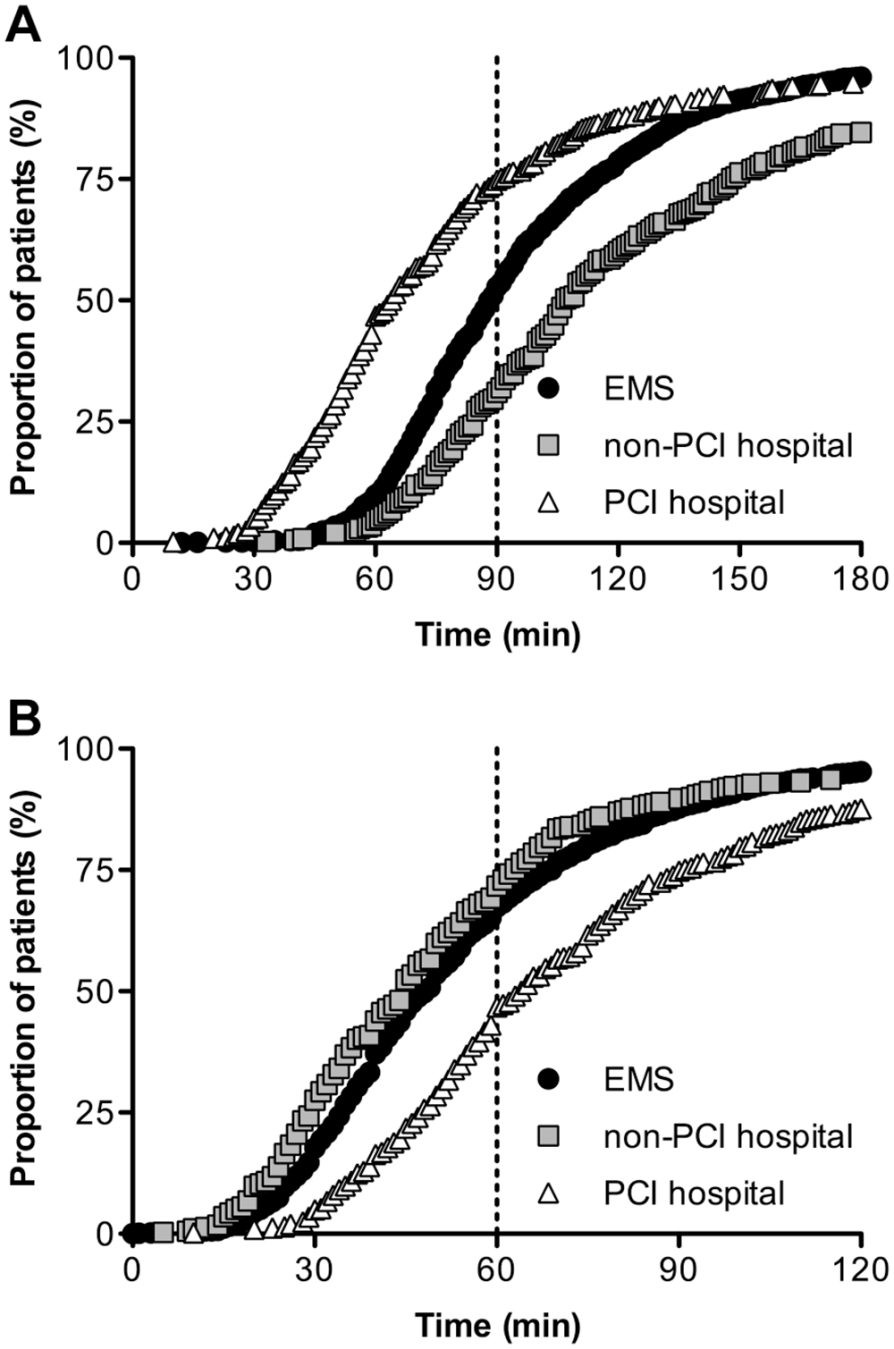
Cumulative frequency distribution of critical time intervals by type of first medical contact. (A) Proportion of patients who were treated within certain contact-to-balloon times with guideline-recommended target time of 90 minutes highlighted with dashed line. (B) Proportion of patients who were treated within certain door-to-balloon times with guideline-recommended target time of 60 minutes highlighted with dashed line. EMS indicates emergency medical services, PCI indicates percutaneous coronary intervention.

Table 2 shows critical time intervals of patients receiving coronary angiography stratified by type of FMC. A longer symptom-to-contact time accounted for the majority of difference in symptom-to-balloon time between groups. However, a longer contact-to-door time in patients presenting to non-PCI-capable hospitals also contributed to overall differences in symptom-to-balloon time. Contact-to-balloon time was shortest for patients presenting to PCI-capable hospitals (65 minutes; IQR 48–91) and longest for patients presenting to non-PCI-capable hospitals (107 minutes; IQR 85–148, p < 0.001). The cumulative frequency distributions of contact-to-balloon and door-to-balloon times are shown in Fig 2.

**Table 2:** Critical time intervals of patients with coronary angiography.

Parameter	Total (n = 3188)	EMS (n = 2168)	Non-PCI hospital (n = 559)	PCI hospital (n = 461)	P Value
**Symptom-to-contact time (min)**	120 (32–330)	75 (30–240)	180 (75–600)	180 (60–480)	<0.001
**Symptom-to-contact time** **≤2 hours**	58.2%	65.6%	40.2%	44.4%	<0.001
**Contact-to-door time (min)**	40 (30–55)	38 (30–48)	60 (45–101)	—	<0.001
**Symptom-to-door**^a^ **time (min)**	145 (76–360)	117 (71–270)	282 (160–725)	180 (60–480)	<0.001
**Door-to-balloon**^b^ **time (min)**	50 (35–72)	49 (35–70)	45 (30–63)	65 (48–91)	<0.001
**Contact-to-balloon time (min)**	88 (69–116)	89 (72–115)	107 (85–148)	65 (48–91)	<0.001
**Symptom-to-balloon time (min)**	201 (130–414)	174 (123–320)	328 (202–765)	254 (138–639)	<0.001

Due to some missing data, time intervals do not always add up. Median and interquartile range or frequency (%), P values are from Kruskal-Wallis test.

^a^ defined as door of the first hospital entered

^b^ defined as door of the interventional hospital

Contact-to-balloon time of less than 90 minutes was achieved in 51.8% of patients. Door-to-balloon time of less than 60 minutes was achieved in 62.2% of patients. Achievement of these treatment goals differed significantly among the three groups. Contact-to-balloon time of less than 90 minutes was achieved in 51.0% of patients who presented to EMS, 29.8% of patients who presented to non-PCI-capable hospitals, and 73.8% of patients who presented to PCI-capable hospitals (p < 0.001). Door-to-balloon time of less than 60 minutes was achieved in 65.1% in the EMS group, 69.2% in the non-PCI-capable hospital group, and 43.1% in the PCI-capable hospital group (p < 0.001).

### Angiographic and procedural results

**Fig 3:**
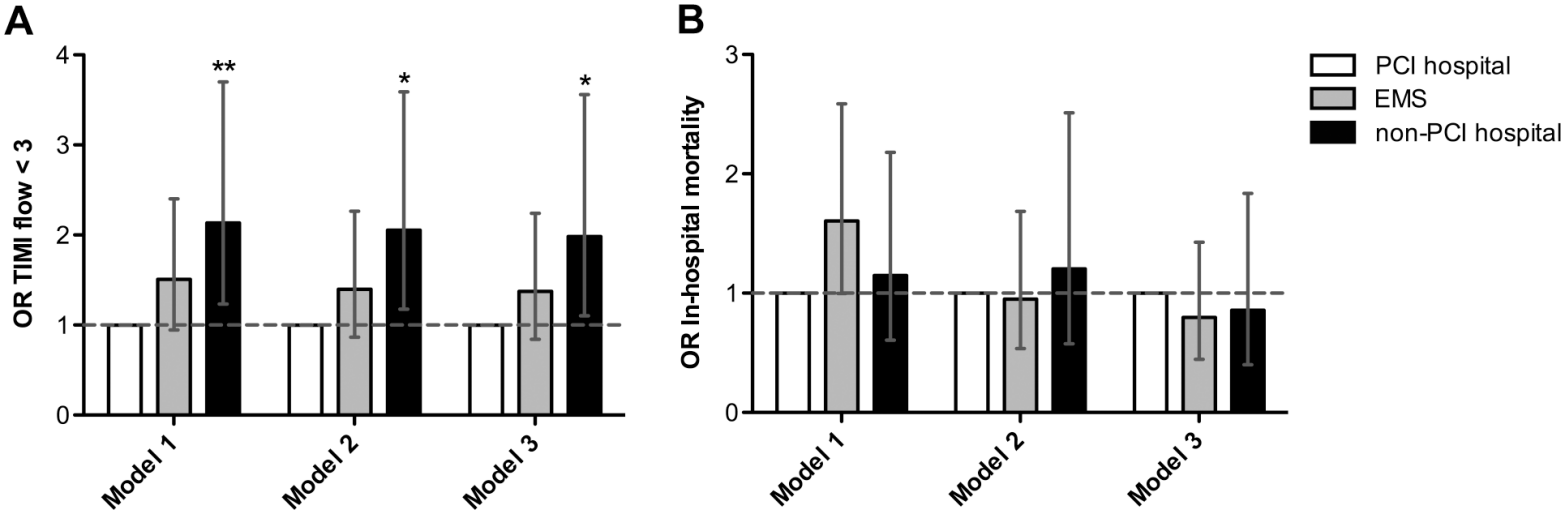
Logistic regression analyses of type of first medical contact (FMC) on procedural results and in-hospital mortality. Odds ratios (OR) of (*A*) TIMI flow < 3 and (*B*) in-hospital mortality by type of FMC unadjusted (Model 1), adjusted for baseline characteristics (age, gender, history of stroke, heart rate > 100/min, systolic blood pressure < 100 mmHg, cardiogenic shock) and symptom-to-contact time (Model 2), and adjusted as Model 2 plus contact-to-balloon time (Model 3). OR are indicated by bars, with the lines representing corresponding 95% confidence intervals. The horizontal line indicates no difference between type of FMC. * p < 0.05, ** p < 0.01.

Angiographic and procedural results are summarized in Table 3. TIMI flow grade 3 after procedure in the infarct related vessel was achieved more often in the PCI-capable hospital group (84.4%) compared to the EMS (75.7%) or non-PCI-capable hospital group (70.3%; p < 0.006). When compared to patients presenting to PCI-capable hospitals, patients presenting to EMS had a numerically increased risk of post-procedural TIMI flow grade < 3 (OR 1.51, 95% CI 0.95–2.40, p = 0.09) and patients presenting to non-PCI capable hospitals had a significantly increased risk of post-procedural TIMI flow grade < 3 (OR 2.14, 95% CI 1.23–3.70, p = 0.007). The latter remained significant when adjusting for baseline characteristics (age, gender, history of stroke, heart rate > 100/min, systolic blood pressure < 100 mmHg, cardiogenic shock), symptom-to-contact time and contact-to-balloon time (OR 1.98, 95% CI 1.10–3.56, p = 0.02; Fig 3).

**Table 3:** Angiographic and procedural results.

Parameter	Total (n = 3188)	EMS (n = 2168)	Non-PCI hospital (n = 559)	PCI hospital (n = 461)	P Value
**Infarct related artery—no. (%)**					
**Left anterior descending**	1,239 (38.9)	833 (38.4)	218 (39.0)	188 (40.8)	0.63
**Diagonal branch**	112 (3.5)	70 (3.2)	26 (4.7)	16 (3.5)	0.26
**Left circumflex**	415 (13.0)	302 (13.3)	64 (11.4)	49 (10.6)	0.08
**Posterolateral branch**	75 (2.4)	52 (2.4)	13 (2.3)	10 (2.2)	0.98
**Right coronary**	1,119 (35.1)	768 (35.4)	182 (32.6)	169 (36.7)	0.34
**Venous bypass graft**	39 (1.2)	25 (1.2)	3 (0.5)	11 (2.4)	0.03
**Arterial bypass graft**	9 (0.3)	6 (0.3)	1 (0.2)	2 (0.4)	0.77
**Other vessel branch**	72 (2.3)	54 (2.5)	11 (2.0)	7 (1.5)	0.43
**Missing data**	364 (11.4)	250 (11.5)	84 (15.0)	30 (6.5)	<0.001
**Procedural results—no. (%)**					
**PCI performed**	2,751 (86.3)	1,864 (86.0)	463 (82.8)	424 (92.0)	<0.001
**Stenting performed**	2,456 (77.0)	1,655 (76.3)	402 (71.9)	399 (86.6)	<0.001
**CABG performed**	117 (3.7)	82 (3.8)	18 (3.2)	17 (3.7)	0.86
**TIMI 3 flow after procedure**	2,424 (76.0)	1,642 (75.7)	393 (70.3)	389 (84.4)	0.006

P values are from Fisher’s exact test.

### Clinical results

In-hospital outcomes are shown in Table 4. There were no statistically significant differences in rate of re-infarction, neurologic or bleeding complications, re-intervention of the infarct related coronary artery and length of hospital stay between the different groups. When compared to patients presenting to PCI-capable hospitals, patients presenting to EMS had a numerically increased risk of in-hospital mortality (OR 1.61, 95% CI 1.00–2.57, p = 0.06) whereas patients presenting to non-PCI capable hospitals had no significantly different risk of in-hospital mortality (OR 1.15, 95% CI 0.61–2.18, p = 0.67). The former association in patients presenting to EMS was not present anymore when adjusting for baseline characteristics of patients (OR 0.93, 95% CI 0.53–1.64, p = 0.81, adjusted for age, gender, history of stroke, heart rate > 100/min, systolic blood pressure < 100 mmHg, cardiogenic shock; Fig 3).

**Table 4:** In-hospital outcomes of patients with coronary angiography.

	Total (n = 3188)	EMS (n = 2168)	Non-PCI hospital (n = 559)	PCI hospital (n = 461)	P Value
**Death—no. (%)**	300 (9.4)	226 (10.4)	48 (8.6)	26 (5.6)	0.002^a^
**Re-infarction—no. (%)**	45 (1.4)	35 (1.6)	5 (0.9)	5 (1.1)	0.50^a^
**Neurologic complication—no. (%)**	38 (1.2)	29 (1.3)	7 (1.3)	2 (0.4)	0.22^a^
**Major bleeding—no. (%)**	33 (1.0)	23 (1.1)	6 (1.1)	4 (0.9)	0.90^a^
**Length of hospital stay –median days (interquartile range)**	8 (6–11)	8 (5–11)	8 (5–11)	8 (6–11)	0.07^b^
**Re-intervention at the infarct-related artery—no. (%)**	120 (3.8)	85 (3.9)	14 (2.5)	21 (4.6)	0.17^a^

^a^ Fisher’s exact test;

^b^ Kruskal-Wallis test

### Subgroup analysis

We performed analysis stratified by inclusion period (2006 to 2009 compared to 2010 to 2012) to address potential effects of temporal changes of management and treatment. The type of FMC changed only slightly over time with minor increases in presentation to EMS and PCI capable hospitals, which was of borderline significance (S1 Table, p = 0.04). The overall contact to balloon time significantly decreased by 5 minutes which was mainly driven by the EMS presenting group (S1 Table). We did not observe a statistically significant interaction of inclusion period on the association of type of FMC with outcome (test for interaction p = 0.70 for in-hospital mortality and p = 0.20 for TMI flow grade < III).

Patients presenting early (≤ 2 hours of symptom to contact time) or with cardiogenic shock more frequently were seen by EMS (77.3% and 83.1%) than patients presenting later or without shock (56.6% and 65.5%, both p < 0.001, S2 and S3 Tables). The overall contact to balloon time was significantly shorter in early presenting patients (86 min [68 min; 112 min] versus 90 min [70 min; 120 min], p = 0.03), which was partly driven by patients presenting to EMS but mainly by patients presenting to PCI capable hospitals (S2 Table). The overall contact to balloon time was significantly longer in patients with shock (99 min [77 min; 125 min] versus 87 min [68 min; 114 min], p < 0.001), which was driven by patients presenting to EMS (S3 Table). We did not observe a statistically significant interaction of early presentation or presence of shock on the association of type of FMC with outcome (test for interaction p = 0.87 and p = 0.07 for in-hospital mortality and p = 0.83 and p = 0.51 for TMI flow grade<III).

The type of FMC did not differ significantly by gender and age (≥ 75 years versus < 75 years, S4 and S5 Tables). The overall contact to balloon time was shorter in male and younger patients. There was no significant interaction of gender and age on the association of type of FMC with outcome (all tests for interaction p > 0.20).

## Discussion

We present results from a metropolitan network providing primary PCI to all patients presenting with STEMI with a median contact-to balloon time within targets recommended by current guidelines [5]. The system related delay to reperfusion differed significantly by the site of FMC, with an excess in the median contact-to-balloon time of 24 minutes for patients presenting to field EMS and 42 minutes for patients presenting to non-PCI-capable hospitals compared to patients presenting to a PCI-capable hospital. The rate of post-procedural TIMI flow grade < 3 differed significantly by site of FMC. When adjusting for baseline characteristics and total delay to reperfusion, the risk of TIMI flow grade < 3 was still about 14% higher in patients presenting to non-PCI-capable hospitals compared to patients presenting to PCI-capable hospitals. Differences in in-hospital mortality observed by site of FMC were not significant when adjusting for baseline characteristics of patients.

The majority of STEMI patients in KIM presented to field EMS whereas about a third of patients presented to emergency departments of a hospital, which did not substantially change over the 7 years of observation. Although these figures might depend on local health care structures, similar distributions were reported by other urban STEMI networks [11–15]. Patients presenting to EMS had a more than halved symptom-to-contact time compared to patients who present to a hospital emergency department which most likely is the result of a more severe initial manifestation of STEMI with hemodynamic instability. Accordingly, the unadjusted in-hospital mortality in EMS presenting patients was higher despite short system related delay times which was similarly reported in earlier studies [12, 15–17]. Given the high mortality in unstable STEMI patients despite optimal network conditions including EMS logistics and primary PCI reperfusion there is a clear need for novel strategies to improve outcome [18]. Currently hemodynamic mechanical support devices such as extracorporeal membrane oxygenation (ECMO) are increasingly applied in STEMI patients with cardiogenic shock. So far randomized trials are lacking but observational studies suggest an improvement in survival associated with ECMO use [19].

The system related delay to reperfusion differed significantly by the site of FMC and the question rises whether this translates into patient outcome. The rate of in-hospital mortality did not differ significantly by site of FMC when adjusted for baseline severity of STEMI. A body of literature shows a correlation between treatment delay and mortality in STEMI patients. In a large Swedish registry including 11,400 patients a significant effect of delay time on 1-year mortality was detectable beyond a cut-off of 1 hour, with each additional 30 minute delay in contact-to-balloon time associated with an estimated 6% increase in 1-year mortality [20]. Notably, our findings must be interpreted in the setting of a guideline conform STEMI network with primary PCI amenable to all patients and overall small absolute delay times which were clearly shorter than those reported by other networks [12–14, 16, 21–23]. In line with our findings recent studies also did not observe an improvement in outcome associated with a further reduction in door to balloon time [24, 25]. A pooled analysis of randomized trials showed a linear association between treatment delay and outcome for patients treated with fibrinolysis but not for patients treated with primary PCI which might be one explanation for our findings [26]. Another explanation is that the small differences in delay associated with site of FMC in combination with a short absolute system related delay in our network might not relevantly impact short-term mortality. However, we observed differences in the angiographic reperfusion success by type of FMC with the highest rate of post-procedural TMI flow grade < 3 in patients presenting to non-PCI-capable compared to PCI-capable hospitals. This association persisted after adjustment for baseline characteristics and was only marginally attenuated when adjusting for total delay to reperfusion. Mechanisms underlying this observation are unclear so far, and might very well be related to patient characteristics incidentally differing by referral areas of non-PCI and PCI-capable hospitals in Cologne. For instance, patients presenting to non-PCI capable hospitals were older than those presenting to PCI-capable hospitals. In elderly STEMI patients symptom-to-contact time might be underestimated due to atypical symptoms [27] and higher comorbidity might also negatively affect success of PCI [28]. Apart from this, albeit our system-related delay in patients presenting to non-PCI capable hospitals could be further reduced as has been demonstrated in other STEMI networks, a longer system related delay in patients presenting to non-PCI capable hospitals will always persist due to inter-hospital transfer logistics [29, 30]. Accordingly, in order to further reduce the total ischemic time burden in STEMI patients community campaigns are needed to increase the rate of EMS presentation as the fastest reperfusion pathway and to increase the awareness of signs of myocardial infarction so that patients seek medical attention earlier. A recent study demonstrated the feasibility and effectivity of a mass media campaign on warning signs suggestive of acute coronary syndrome and appropriate actions which lead to significantly reduced pre-hospital delay times [31].

The KIM registry did not include STEMI patients who were already hospitalized or who were diagnosed but were not eligible for coronary angiography for instance in palliative care settings. Also, patients with sudden cardiac death who were dead on arrival of EMS and who might initially have had a STEMI were not registered in our database. Therefore, our data probably underestimate true STEMI mortality in the population of the city of Cologne but might be more comparable to other registries using similar inclusion criteria. Further, the documentation of most data of the registry was carried out by primary care takers at EMS and hospitals. Thus, completeness and quality of data depend on the motivation of participating physicians and trends to artificially improve their own treatment outcomes cannot be fully excluded. So far, we did not have an audit to control validity of the KIM registry. Characterization of patients was limited due to the fact that only data for quality control purposes were collected. This also means that we have no sophisticated measures of comorbidity and outcome such as size of infarction or left-ventricular function available and no long-term data on mortality or heart failure, which might be more sensitive to detect effects of treatment delays. Finally, our results must be seen in the setting of a well established metropolitan STEMI network with obligatory primary PCI available for all patients and cannot be transferred to other systems of STEMI care for example in a rural setting since system related delay clearly correlated with the distance of transfer or systems using fibrinolysis treatment [32].

## Conclusions

In conclusion, within this guideline conform metropolitan STEMI network a substantial proportion of patients self-presented directly to emergency departments of hospitals which is of concern since it is associated with a delay to reperfusion for all patients presenting to non-PCI capable hospitals. We did not observe a significant effect of the apparent treatment delay related to distinct FMC presentation on in-hospital mortality and procedural success under the overall optimized network logistics giving strong support to existing guidelines. However, we cannot exclude minor effects on intermediate outcomes such as infarction size, which might affect long-term outcome of patients. Our study demonstrates long-term feasibility and effectivity of a guideline-conform metropolitan STEMI-network providing primary PCI within recommended times to virtually all patients. Optimizing logistics within a STEMI network can adjust short-term outcomes for different types of first medical contact despite persisting small treatment delays.

## Supporting Information

S1 Fig Triage for patients presenting with STEMI in KIM. (TIF)

S1 Table Temporal trend of type of first medical contact and associated contact-to-balloon and symptom-to-contact time. (DOCX)

S2 Table Type of first medical contact and associated contact-to-balloon times in early (≤2 hours of symptom to contact time) and delayed presenting patients. (DOCX)

S3 Table Type of first medical contact and associated contact-to-balloon time and in-hospital mortality by cardiogenic shock. (DOCX)

S4 Table Type of first medical contact and associated contact-to-balloon time in men and women. (DOCX)

S5 Table Type of first medical contact and associated contact-to-balloon time by age. (DOCX)
