# Supplementary figures and images for: Impact of the Type of First Medical Contact within a Guideline-Conform ST-Elevation Myocardial Infarction Network: A Prospective Observational Registry Study
Source: PLoS One. 2016 Jun 3;11(6):e0156769. doi: 10.1371/journal.pone.0156769 (PMC4892676; doi:10.1371/journal.pone.0156769)

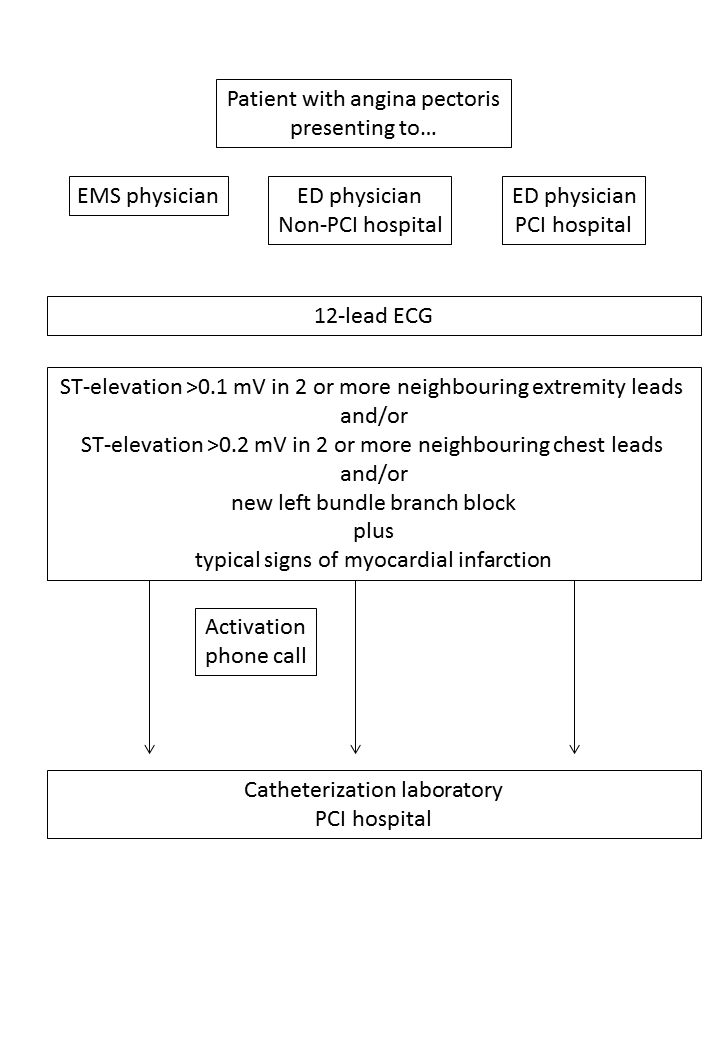

Supplement: S1 Fig — (TIF) [file pone.0156769.s001.tif]
