# Supplementary material for: Impact of the Type of First Medical Contact within a Guideline-Conform ST-Elevation Myocardial Infarction Network: A Prospective Observational Registry Study
Source: PLoS One. 2016 Jun 3;11(6):e0156769. doi: 10.1371/journal.pone.0156769 (PMC4892676; doi:10.1371/journal.pone.0156769)
Supplement: S1 Table — (DOCX) [file pone.0156769.s002.docx]

|  |  | **Inclusion period** | |  |
| --- | --- | --- | --- | --- |
|  |  | **2006-2009** | **2010-2012** | **p-value** |
| **Type of FMC** | **EMS** | 67.5% | 68.6% | 0.04 |
|  | **non-PCI capable hosp.** | 19.0% | 15.9% |  |
|  | **PCI capable hosp.** | 13.5% | 15.5% |  |
| **C2B time** | **all** | 90 (71; 118) | 85 (67; 114) | 0.001 |
|  | **in EMS** | 91 (74; 115) | 85 (69; 113) | 0.004 |
|  | **in non-PCI capable hospitals** | 107 (85; 148) | 107 (85; 150) | 0.84 |
|  | **in PCI capable hospitals** | 66 (51; 90) | 60 (44; 92) | 0.19 |
| **S2C time** | **all** | 120 (40; 300) | 120 (30; 360) | 0.63 |
|  | **in EMS** | 73 (30; 195) | 75 (30; 240) | 1 |
|  | **in non-PCI capable hospitals** | 180 (60; 540) | 180 (90; 720) | 0.55 |
|  | **in PCI capable hospitals** | 180 (78; 480) | 150 (60; 480) | 0.11 |

FMC: first medical contact, EMS: emergency medical system, PCI: percutaneous coronary intervention, C2B: contact to balloon, S2C: symptom to contact, median and quartiles
