# Supplementary material for: Impact of the Type of First Medical Contact within a Guideline-Conform ST-Elevation Myocardial Infarction Network: A Prospective Observational Registry Study
Source: PLoS One. 2016 Jun 3;11(6):e0156769. doi: 10.1371/journal.pone.0156769 (PMC4892676; doi:10.1371/journal.pone.0156769)
Supplement: S2 Table — (DOCX) [file pone.0156769.s003.docx]

|  |  | **Symptom to contact time** | |  |
| --- | --- | --- | --- | --- |
|  |  | **<2 hours** | **>2 hours** | **p-value** |
| **Type of FMC** | **EMS** | 77.3% | 56.6% | <0.001 |
|  | **non-PCI capable hosp.** | 11.7% | 24.1% |  |
|  | **PCI capable hosp.** | 11.0% | 19.3% |  |
| **C2B time** | **all** | 86 (68;112) | 90 (70; 120) | 0.03 |
|  | **in EMS** | 88 (71; 112) | 90 (73; 117) | 0.12 |
|  | **in non-PCI capable hospitals** | 110 (83; 142) | 106 (85; 153) | 0.68 |
|  | **in PCI capable hospitals** | 60 (46; 80) | 73 (49; 100) | 0.007 |

FMC: first medical contact, EMS: emergency medical system, PCI: percutaneous coronary intervention, C2B: contact to balloon, median and quartiles, median and quartiles
