# Supplementary material for: Impact of the Type of First Medical Contact within a Guideline-Conform ST-Elevation Myocardial Infarction Network: A Prospective Observational Registry Study
Source: PLoS One. 2016 Jun 3;11(6):e0156769. doi: 10.1371/journal.pone.0156769 (PMC4892676; doi:10.1371/journal.pone.0156769)
Supplement: S3 Table — (DOCX) [file pone.0156769.s004.docx]

|  |  | **Cardiogenic shock** | |  |
| --- | --- | --- | --- | --- |
|  |  | **yes** | **no** | **p-value** |
| **Type of FMC** | **EMS** | 83.1% | 65.5% | <0.001 |
|  | **non-PCI capable hospital** | 9.4% | 18.9% |  |
|  | **PCI capable hospital** | 7.6% | 15.6% |  |
| **C2B time** | **all** | 99 (77; 125) | 87 (68; 114) | <0.001 |
|  | **in EMS** | 102 (79; 125) | 87 (70; 111) | <0.001 |
|  | **in non-PCI capable hospitals** | 115 (80; 134) | 107 (85; 150) | 0.685 |
|  | **in PCI capable hospitals** | 60 (48; 81) | 65 (48; 93) | 0.645 |
| **In-hospital mortality** |  | 44.8% | 4.6% | <0.001 |

FMC: first medical contact, EMS: emergency medical system, PCI: percutaneous coronary intervention, C2B: contact to balloon Median and quartiles
