# Supplementary material for: Impact of the Type of First Medical Contact within a Guideline-Conform ST-Elevation Myocardial Infarction Network: A Prospective Observational Registry Study
Source: PLoS One. 2016 Jun 3;11(6):e0156769. doi: 10.1371/journal.pone.0156769 (PMC4892676; doi:10.1371/journal.pone.0156769)
Supplement: S4 Table — (DOCX) [file pone.0156769.s005.docx]

|  |  | **Men** | **Women** | **p-value** |
| --- | --- | --- | --- | --- |
| **Type of FMC** | **EMS** | 68.4% | 66.8% | 0.15 |
|  | **non-PCI capable hospital** | 16.8% | 19.7% |  |
|  | **PCI capable hospital** | 14.8% | 13.5% |  |
| **C2B time** |  | 87 (68; 115) | 90 (73; 120) | 0.01 |

FMC: first medical contact, EMS: emergency medical system, PCI: percutaneous coronary intervention, C2B: contact to balloon, median and quartiles
